# Resuscitation fluid practices in Brazilian intensive care units: a secondary analysis of Fluid-TRIPS

**DOI:** 10.5935/0103-507X.20210028

**Published:** 2021

**Authors:** Flavio Geraldo Rezende de Freitas, Naomi Hammond, Yang Li, Luciano Cesar Pontes de Azevedo, Alexandre Biasi Cavalcanti, Leandro Taniguchi, André Gobatto, André Miguel Japiassú, Antonio Tonete Bafi, Bruno Franco Mazza, Danilo Teixeira Noritomi, Felipe Dal-Pizzol, Fernando Bozza, Jorge Ibrahin Figueira Salluh, Glauco Adrieno Westphal, Márcio Soares, Murillo Santucci César de Assunção, Thiago Lisboa, Suzana Margarete Ajeje Lobo, Achilles Rohlfs Barbosa, Adriana Fonseca Ventura, Ailson Faria de Souza, Alexandre Francisco Silva, Alexandre Toledo, Aline Reis, Allan Cembranel, Alvaro Rea Neto, Ana Lúcia Gut, Ana Patricia Pierre Justo, Ana Paula Santos, André Campos D. de Albuquerque, André Scazufka, Antonio Babo Rodrigues, Bruno Bonaccorsi Fernandino, Bruno Goncalves Silva, Bruno Sarno Vidal, Bruno Valle Pinheiro, Bruno Vilela Costa Pinto, Carlos Augusto Ramos Feijo, Carlos de Abreu Filho, Carlos Eduardo da Costa Nunes Bosso, Carlos Eduardo Nassif Moreira, Carlos Henrique Ferreira Ramos, Carmen Tavares, Cidamaiá Arantes, Cintia Grion, Ciro Leite Mendes, Claudio Kmohan, Claudio Piras, Cristine Pilati Pileggi Castro, Cyntia Lins, Daniel Beraldo, Daniel Fontes, Daniela Boni, Débora Castiglioni, Denise de Moraes Paisani, Durval Ferreira Fonseca Pedroso, Ederson Roberto Mattos, Edgar de Brito Sobrinho, Edgar M. V. Troncoso, Edison Moraes Rodrigues Filho, Eduardo Enrico Ferrari Nogueira, Eduardo Leme Ferreira, Eduardo Souza Pacheco, Euzebio Jodar, Evandro L. A. Ferreira, Fabiana Fernandes de Araujo, Fabiana Schuelter Trevisol, Fábio Ferreira Amorim, Fabio Poianas Giannini, Fabrício Primitivo Matos Santos, Fátima Buarque, Felipe Gallego Lima, Fernando Antonio Alvares da Costa, Fernando Cesar dos Anjos Sad, Fernando G. Aranha, Fernando Ganem, Flavio Callil, Francisco Flávio Costa Filho, Frederico Toledo Campo Dall´Arto, Geovani Moreno, Gilberto Friedman, Giulliana Martines Moralez, Guilherme Abdalla da Silva, Guilherme Costa, Guilherme Silva Cavalcanti, Guilherme Silva Cavalcanti, Gustavo Navarro Betônico, Gustavo Navarro Betônico, Hélder Reis, Helia Beatriz N. Araujo, Helio Anjos Hortiz Júnior, Helio Penna Guimaraes, Hugo Urbano, Israel Maia, Ivan Lopes Santiago Filho, Jamil Farhat Júnior, Janu Rangel Alvarez, Joel Tavares Passos, Jorge Eduardo da Rocha Paranhos, José Aurelio Marques, José Gonçalves Moreira Filho, Jose Neto Andrade, José Onofre de C Sobrinho, Jose Terceiro de Paiva Bezerra, Juliana Apolônio Alves, Juliana Ferreira, Jussara Gomes, Karina Midori Sato, Karine Gerent, Kathia Margarida Costa Teixeira, Katia Aparecida Pessoa Conde, Laércia Ferreira Martins, Lanese Figueirêdo, Leila Rezegue, Leonardo Tcherniacovsk, Leone Oliveira Ferraz, Liane Cavalcante, Ligia Rabelo, Lilian Miilher, Lisiane Garcia, Luana Tannous, Ludhmila Abrahão Hajjar, Luís Eduardo Miranda Paciência, Luiz Monteiro da Cruz Neto, Macia Valeria Bley, Marcelo Ferreira Sousa, Marcelo Lourencini Puga, Marcelo Luz Pereira Romano, Marciano Nobrega, Marcio Arbex, Márcio Leite Rodrigues, Márcio Osório Guerreiro, Marcone Rocha, Maria Angela Pangoni Alves, Maria Angela Pangoni Alves, Maria Doroti Rosa, Mariza D’Agostino Dias, Miquéias Martins, Mirella de Oliveira, Miriane Melo Silveira Moretti, Mirna Matsui, Octavio Messender, Orlando Luís de Andrade Santarém, Patricio Júnior Henrique da Silveira, Paula Frizera Vassallo, Paulo Antoniazzi, Paulo César Gottardo, Paulo Correia, Paulo Ferreira, Paulo Torres, Pedro Gabrile M. de Barros e Silva, Rafael Foernges, Rafael Gomes, Rafael Moraes, Raimundo Nonato filho, Renato Luis Borba, Renato V Gomes, Ricardo Cordioli, Ricardo Lima, Ricardo Pérez López, Ricardo Rath de Oliveira Gargioni, Richard Rosenblat, Roberta Machado de Souza, Roberto Almeida, Roberto Camargo Narciso, Roberto Marco, Roberto waltrick, Rodrigo Biondi, Rodrigo Figueiredo, Rodrigo Santana Dutra, Roseane Batista, Rouge Felipe, Rubens Sergio da Silva Franco, Sandra Houly, Sara Socorro Faria, Sergio Felix Pinto, Sergio Luzzi, Sergio Sant’ana, Sergio Sonego Fernandes, Sérgio Yamada, Sérgio Zajac, Sidiner Mesquita Vaz, Silvia Aparecida Bezerra Bezerra, Tatiana Bueno Tardivo Farhat, Thiago Martins Santos, Tiago Smith, Ulysses V. A. Silva, Valnei Bento Damasceno, Vandack Nobre, Vicente Cés de Souza Dantas, Vivian Menezes Irineu, Viviane Bogado, Wagner Nedel, Walther Campos Filho, Weidson Dantas, William Viana, Wilson de Oliveira Filho, Wilson Martins Delgadinho, Simon Finfer, Flavia Ribeiro Machado

**Affiliations:** 1 Discipline of Anesthesiology, Pain and Intensive Care, Universidade Federal de São Paulo - São Paulo (SP), Brazil.; 2 Critical Care and Trauma Division, The George Institute for Global Health - Sydney, Austrália.; 3 Hospital Sírio-Libanês - São Paulo (SP), Brazil.; 4 Hospital das Clínicas, Faculdade de Medicina, Universidade de São Paulo - São Paulo (SP), Brazil.; 5 Instituto de Pesquisa, HCor - Hospital do Coração - São Paulo (SP), Brazil.; 6 Instituto de Pesquisa Clínica Evandro Chagas, Fundação Oswaldo Cruz - Rio de Janeiro (RJ), Brazil.; 7 Hospital de Clínicas Mário Lioni - Rio de Janeiro (RJ), Brazil.; 8 Hospital do Rim e Hipertensão, Fundação Oswaldo Ramos - São Paulo (SP), Brazil.; 9 Hospital Samaritano - São Paulo (SP), Brazil.; 10 Hospital Paulistano - São Paulo (SP), Brazil.; 11 Hospital São José - Criciúma (SC), Brazil.; 12 Instituto D’Or de Ensino e Pesquisa - Rio de Janeiro (RJ), Brazil.; 13 Hospital Municipal São José - Joinville (SC), Brazil.; 14 Hospital Israelita Albert Einstein - São Paulo (SP), Brazil.; 15 Hospital Santa Rita - Porto Alegre (RS), Brazil.; 16 Hospital de Base, Faculdade de Medicina de São Jose do Rio Preto - São José do Rio Preto (SP), Brazil.; 17 Hospital Unimed de Belo Horizonte - Belo Horizonte (MG), Brazil.; 18 Hospital Santa Lúcia - Divinópolis (MG), Brazil.; 19 Hospital Evangélico de Sorocaba - Sorocaba (SP), Brazil.; 20 Hospital PIO XII - São José dos Campos (SP), Brazil.; 21 Hospital São Camilo Pompéia - São Paulo (SP), Brazil.; 22 Hospital Alvorada Brasília - Brasília (DF), Brazil.; 23 Hospital Ecoville - Curitiba (PR), Brazil.; 24 Hospital do Trabalhador - Curitiba (PR), Brazil.; 25 Hospital Vita Batel - Curitiba (PR), Brazil.; 26 Hospital das Clínicas, Faculdade de Medicina de Botucatu, Universidade Estadual “Júlio de Mesquita Filho” - Botucatu (SP), Brazil.; 27 Hospital Geral Dr. César Cals - Fortaleza (CE), Brazil.; 28 Hospital Copa D’Or - Rio de Janeiro (RJ), Brazil.; 29 Associação Beneficente Hospital Unimar - Marília (SP), Brazil.; 30 Hospital Casa de Saúde de Santos - Santos (SP), Brazil.; 31 Hospital Estadual Getúlio Vargas - Rio de Janeiro (RJ), Brazil.; 32 Hospital e Maternidade Otaviano Neves - Belo Horizonte (MG), Brazil.; 33 Instituto Estadual do Cérebro Paulo Niemeyer- Rio de Janeiro (RJ), Brazil.; 34 Hospital Universitário, Universidade Federal de Juiz de Fora- Juiz de Fora (MG), Brazil.; 35 Lifecenter - Belo Horizonte (MG), Brazil.; 36 Hospital Geral de Fortaleza - Fortaleza (CE), Brazil.; 37 Hospital Municipal Dr. Moysés Deutsch (M’Boi Mirim) - São Paulo (SP), Brazil.; 38 Santa Casa de Misericórdia de Presidente Prudente - Presidente Prudente (SP), Brazil.; 39 Hospital 9 de julho - São Paulo (SP), Brazil.; 40 Hospital Estadual Rocha Faria - Rio de Janeiro (RJ), Brazil.; 41 Hospital Municipal Santa Isabel - João Pessoa (PA), Brazil.; 42 Hospital e Maternidade Municipal Dr. Odelmo Leão Carneiro - Uberlândia (MG), Brazil.; 43 Hospital Evangélico de Londrina - Londrina (PR), Brazil.; 44 Universidade Estadual de Londrina - Londrina (SC), Brazil.; 45 Hospital Samaritano João Pessoa - João Pessoa (PB), Brazil.; 46 Hospital de Caridade Astrogildo de Azevedo - Santa Maria (RS), Brazil.; 47 Vitória Apart Hospital - Vitória (ES), Brazil.; 48 Instituto de Ortopedia e Traumatologia - Passo Fundo (RS), Brazil.; 49 Hospital do Subúrbio - Salvador (BA), Brazil.; 50 Hospital Renascentista - Pouso Alegre (MG), Brazil.; 51 Hospital Felício Rocho - Belo Horizonte (MG), Brazil.; 52 Hospital Municipal Irmã Dulce - Praia Grande (SP), Brazil.; 53 Hospital Universitário Júlio Müller, Universidade Federal do Mato Grosso - Cuiabá (MT), Brazil.; 54 Hospital de Urgência - Goiânia (GO), Brazil.; 55 Hospital Geral de Goiânia - Goiânia (GO), Brazil.; 56 Fundação Doutor Amaral Carvalho - Jaú (SP), Brazil.; 57 Hospital Adventista de Belém - Belém (PA), Brazil.; 58 Hospital Santa Juliana - Rio Branco (AC), Brazil.; 59 Hospital Dom Vicente Scherer - Porto Alegre (RS), Brazil.; 60 Hospital Primavera - Aracaju (SE), Brazil.; 61 Hospital Carlos da Silva Lacaz - Francisco Morato (SP), Brazil.; 62 Hospital Escola, Faculdade de Medicina de Jundiaí - Jundiaí (SP), Brazil.; 63 Hospital Sepaco - São Paulo (SP), Brazil.; 64 Hospital Professor Edmundo Vasconcelos - São Paulo (SP), Brazil.; 65 Hospital Paulo Sacramento - Jundiaí (SP), Brazil.; 66 Clínica Dom Rodrigo - João Pessoa (PA), Brazil.; 67 Complexo Hospitalar Ortotrauma de Mangabeira - Fortaleza (CE), Brazil.; 68 Hospital Nossa Senhora da Conceição - Tubarão (SC), Brazil.; 69 Hospital Regional de Samambaia - Brasília (DF), Brazil.; 70 Hospital São Camilo Ipiranga - São Paulo (SP), Brazil.; 71 Hospital da Restauração - Recife (PE), Brazil.; 72 Instituto do Coração, Hospital das Clínicas, Faculdade de Medicina, Universidade de São Paulo - São Paulo (SP), Brazil.; 73 Hospital Santa Rita - São Paulo (SP), Brazil.; 74 Hospital Estadual Jayme Santos Neves - Recife (PE), Brazil.; 75 Hospital SOS Cárdio - Florianópolis (SC), Brazil.; 76 Hospital da Luz Vila Mariana - São Paulo (SP), Brazil.; 77 Hospital Maternidade e Pronto-Socorro Santa Luci - Poços de Caldas (MG), Brazil.; 78 Santa Casa de Misericórdia de Vitória da Conquista - Vitória da Conquista (BA), Brazil.; 79 Santa Casa de Misericórdia de Porto Alegre - Porto Alegre (RS), Brazil.; 80 Hospital São Francisco de Assis - Porto Real (RJ), Brazil.; 81 Hospital Memorial São José - Recife (PE), Brazil.; 82 Hospital Regional de Jundiaí - Jundiaí (SP), Brazil.; 83 Hospital Universitário, Faculdade de Medicina de Jundiaí - Jundiaí (SP), Brazil.; 84 Hospital Regional de Presidente Prudente - Presidente Prudente (SP), Brazil.; 85 Santa Casa de Misericórdia de Assis - Assis (SP), Brazil.; 86 Hospital de Clínicas Gaspar Vianna - Belém (PA), Brazil.; 87 Hospital do Coração do Brasil - Brasília (DF), Brazil.; 88 Hospital Hélio Anjos Ortiz - Curitibanos (SC), Brazil.; 89 Hospital Vila da Serra - Belo Horizonte (MG), Brazil.; 90 Hospital Nereu Ramos - Florianópolis (SC), Brazil.; 91 Hospital Santa Maria Intensibarra - Barra Mansa (RJ), Brazil.; 92 Santa Casa de Misericórdia de Santo Amaro - São Paulo (SP), Brazil.; 93 Santa Casa de Caridade de Don Pedrito - Dom Pedrito (RS), Brazil.; 94 Santa Casa de Misericórdia de Santana do Livramento - Santana do Livramento (RS), Brazil.; 95 Hospital Unimed de Macaé - Macaé (RJ), Brazil.; 96 Hospital Municipal Pedro II - Rio de Janeiro (RJ), Brazil.; 97 Hospital Federal dos Servidores do Estado - Rio de Janeiro (RJ), Brazil.; 98 Hospital São Mateus - Fortaleza (CE), Brazil.; 99 Hospital IBR - Vitória da Conquista (BA), Brazil.; 100 Hospital Uniclinic - Fortaleza (CE), Brazil.; 101 Santa Casa de Misericórdia de Paraguaçu Paulista - Paraguaçu Paulista (SP), Brazil.; 102 Associação Hospitalar Beneficente São Vicente de Paulo - Passo Fundo (RS), Brazil.; 103 Hospital do Servidor Público Municipal de São Paulo - São Paulo (SP), Brazil.; 104 Hospital Santa Isabel - Blumenau (SC), Brazil.; 105 Hospital Municipal Dr Jose Soares Hungria - São Paulo (SP), Brazil.; 106 Hospital Fernandes Távora - Fortaleza (CE), Brazil.; 107 Hospital Distrital Evandro Ayres de Moura - Fortaleza (CE), Brazil.; 108 Hospital Saúde da Mulher - Belém (PA), Brazil.; 109 Hospital Estadual de Urgência e Emergência de Vitória - Vitória (ES), Brazil.; 110 Samur - Vitória da Conquista (BA), Brazil.; 111 Hospital e Pronto-Socorro 28 de Agosto - Manaus (AM), Brazil.; 112 Hospital Assunção - São Bernardo do Campo (SP), Brazil.; 113 Hospital Universitário de Santa Maria - Santa Maria (RS), Brazil.; 114 Hospital Universitário Cajuru - Curitiba (PR), Brazil.; 115 Instituto do Câncer do Estado de São Paulo - São Paulo (SP), Brazil.; 116 Hospital Unimed de Limeira - Limeira (SP), Brazil.; 117 Hospital Amecor - Cuiabá (MT), Brazil.; 118 Santa Casa de Caridade de Diamantina - Diamantina (MG), Brazil.; 119 Hospital das Clínicas, Faculdade Ribeirão Preto, Universidade de São Paulo - Ribeirão Preto (SP), Brazil.; 120 HCor - Hospital do Coração - São Paulo (SP), Brazil.; 121 Hospital Goiânia Leste - Goiânia (GO), Brazil.; 122 Hospital Ortopédico - Goiânia (GO), Brazil.; 123 Hospital Santa Maria - Goiânia (GO), Brazil.; 124 Hospital Municipal Dr. Munir Rafful - Volta Redonda (RJ), Brazil.; 125 Hospital Jardim Amália - Volta Redonda (RJ), Brazil.; 126 Hospital Madre Regina Prottman - Santa Tereza (ES), Brazil.; 127 Hospital Universitário São Francisco de Paula, Universidade Católica de Pelotas - Pelotas (RS), Brazil.; 128 Hospital São Joao de Deus - Divinópolis (MG), Brazil.; 129 Hospital Nossa Senhora Monte Serrat - Salto (SP), Brazil.; 130 Hospital Unimed Salto - Salto (SP), Brazil.; 131 Hospital Moinhos de Vento - Porto Alegre (RS), Brazil.; 132 Hospital Geral de Vitória da Conquista - Vitória da Conquista (BA), Brazil.; 133 Hospital Marcelino Champagnat - Curitiba (PR), Brazil.; 134 Hospital São Lucas, Pontifícia Universidade Católica do Rio Grande do Sul - Porto Alegre (RS), Brazil.; 135 Hospital Universitário, Universidade Federal da Grande Dourados - Dourados (GO), Brazil.; 136 Hospital Português - Salvador (BA), Brazil.; 137 Hospital Brigadeiro - São Paulo (SP), Brazil.; 138 Hospital Regional de Sousa - Sousa (PB), Brazil.; 139 Hospital das Clínicas, Universidade Federal do Espírito Santo - Vitória (ES), Brazil.; 140 Santa Casa de Misericórdia de Ribeirão Preto - Ribeirão Preto (SP), Brazil.; 141 Hospital Universitário Lauro Wanderley - João Pessoa (PB), Brazil.; 142 Santa Casa de Belo Horizonte - Belo Horizonte (MG), Brazil.; 143 Hospital Adventista de Manaus - Manaus (AM), Brazil.; 144 Santa Casa Maringá, Universidade Estadual Maringá - Maringá (PR), Brazil.; 145 Hospital Total Cor - Rio de Janeiro (RJ), Brazil.; 146 Hospital Universitário, Universidade de Santa Cruz do Sul - Santa Cruz do Sul (RS), Brazil.; 147 Hospital Dom Hélder - Cabo (PE), Brazil.; 148 Hospital das Clínicas de Porto Alegre, Universidade Federal do Rio Grande do Sul - Porto Alegre (RS), Brazil.; 149 Hospital Anis Rassi - Goiânia (GO), Brazil.; 150 Instituto de Infectologia Emílio Ribas II - São Paulo (SP), Brazil.; 151 Hospital Unimed Rio de Janeiro - Rio de Janeiro (RJ), Brazil.; 152 Hospital Alemão Oswaldo Cruz - São Paulo (SP), Brazil.; 153 Hospital Samaritano Rio de Janeiro - Rio de Janeiro (RJ), Brazil.; 154 São Bernardo Apart Hospital - Colatina (ES), Brazil.; 155 Hospital Nossa Senhora dos Prazeres - Lages (SC), Brazil.; 156 Hospital Unimed ABC - São Bernardo do Campo (SP), Brazil.; 157 Hospital Municipal de Paracatu - Paracatu (MG), Brazil.; 158 Hospital Municipal Padre Germano Lauck - Foz do Iguaçu (RS), Brazil.; 159 Hospital Santa Helena - São Paulo (SP), Brazil.; 160 Hospital Santa Izabel - São Paulo (SP), Brazil.; 161 Santa Casa de Misericórdia de São Paulo - São Paulo (SP), Brazil.; 162 Hospital Tereza Ramos - Lages (SC), Brazil.; 163 Hospital Alvorada Taguatinga - Brasília (DF), Brazil.; 164 Hospital Maternidade São José - Colatina (ES), Brazil.; 165 Hospital Universitário Ciências Médicas, Fundação Educacional Lucas Machado - Belo Horizonte (MG), Brazil.; 166 Santa Casa de Belém do Pará - Belém (PA), Brazil.; 167 Instituto Nacional de Cardiologia - Rio de Janeiro (RJ), Brazil.; 168 Hospital Novo Atibaia - Atibaia (SP), Brazil.; 169 BP - A Beneficência Portuguesa de São Paulo - São Paulo (SP), Brazil.; 170 Hospital Universitário Maria Aparecida Pedrossian, Universidade Federal de Mato Grosso do Sul - Campo Grande (MS), Brazil.; 171 Hospital do Servidor Público Estadual “Francisco Morato de Oliveira” - São Paulo (SP), Brazil.; 172 Hospital Norte D’Or - Rio de Janeiro (RJ), Brazil.; 173 Hospital Estadual Ipiranga - São Paulo (SP), Brazil.; 174 Hospital Universitário de Maringá, Universidade Estadual de Maringá - Maringá (PR), Brazil.; 175 Albert Sabin Hospital e Maternidade - Juiz de Fora (MG), Brazil.; 176 Casa de Caridade de Carangola - Carangola (MG), Brazil.; 177 Irmandade de Misericórdia de Guaxupé - Guaxupé (MG), Brazil.; 178 Disciplina de Emergências Clínicas, Universidade Estadual de Campinas - Campinas (SP), Brazil.; 179 Hospital São Lucas - Aracaju (SE), Brazil.; 180 Fundação Pio XII- Hospital de Câncer de Barretos - Barretos (SP), Brazil.; 181 Clínica Campo Grande - Campo Grande (MS), Brazil.; 182 Hospital das Clínicas, Universidade Federal de Minas Gerais - Belo Horizonte (MG), Brazil.; 183 Casa de Saúde Santa Lúcia - Rio de Janeiro (RJ), Brazil.; 184 Hospital Regional de Itapetininga São Camilo - São Paulo (SP), Brazil.; 185 Santa Casa de Angra dos Reis - Angra dos Reis (RJ), Brazil.; 186 Grupo Hospitalar Nossa Senhora da Conceição - Porto Alegre (RS), Brazil.; 187 Irmandade Misericórdia Hospital Santa Casa de Monte Alto - Monte Alto (SP), Brazil.; 188 Hospital São Marcos - Recife (PE), Brazil.; 189 Hospital Unimed de Manaus - Manaus (AM), Brazil.; 190 Hospital Universitário Getúlio Vargas, Universidade Federal do Amazonas - Manaus (AM), Brazil.; 191 Casa de Saúde Campinas - Campinas (SP), Brazil.; 192 Hospital e Maternidade Galileo - Valinhos (SP), Brazil.

**Keywords:** Fluid therapy, Critical care, Colloids, Crystalloid solutions, Hemodynamics, Shock, Hidratação, Cuidados críticos, Coloides, Soluções cristaloides, Hemodinâmica, Choque

## Abstract

**Objective:**

To describe fluid resuscitation practices in Brazilian intensive care units and to compare them with those of other countries participating in the Fluid-TRIPS.

**Methods:**

This was a prospective, international, cross-sectional, observational study in a convenience sample of intensive care units in 27 countries (including Brazil) using the Fluid-TRIPS database compiled in 2014. We described the patterns of fluid resuscitation use in Brazil compared with those in other countries and identified the factors associated with fluid choice.

**Results:**

On the study day, 3,214 patients in Brazil and 3,493 patients in other countries were included, of whom 16.1% and 26.8% (p < 0.001) received fluids, respectively. The main indication for fluid resuscitation was impaired perfusion and/or low cardiac output (Brazil: 71.7% *versus* other countries: 56.4%, p < 0.001). In Brazil, the percentage of patients receiving crystalloid solutions was higher (97.7% *versus* 76.8%, p < 0.001), and 0.9% sodium chloride was the most commonly used crystalloid (62.5% *versus* 27.1%, p < 0.001). The multivariable analysis suggested that the albumin levels were associated with the use of both crystalloids and colloids, whereas the type of fluid prescriber was associated with crystalloid use only.

**Conclusion:**

Our results suggest that crystalloids are more frequently used than colloids for fluid resuscitation in Brazil, and this discrepancy in frequencies is higher than that in other countries. Sodium chloride (0.9%) was the crystalloid most commonly prescribed. Serum albumin levels and the type of fluid prescriber were the factors associated with the choice of crystalloids or colloids for fluid resuscitation.

## Abstract (pt)

**Objetivo:**

Descrever as práticas de ressuscitação volêmica em unidades de terapia intensiva brasileiras e compará-las com as de outros países participantes do estudo Fluid-TRIPS.

**Métodos:**

Este foi um estudo observacional transversal, prospectivo e internacional, de uma amostra de conveniência de unidades de terapia intensiva de 27 países (inclusive o Brasil), com utilização da base de dados Fluid-TRIPS compilada em 2014. Descrevemos os padrões de ressuscitação volêmica utilizados no Brasil em comparação com os de outros países e identificamos os fatores associados com a escolha dos fluidos.

**Resultados:**

No dia do estudo, foram incluídos 3.214 pacientes do Brasil e 3.493 pacientes de outros países, dos quais, respectivamente, 16,1% e 26,8% (p < 0,001) receberam fluidos. A principal indicação para ressuscitação volêmica foi comprometimento da perfusão e/ou baixo débito cardíaco (Brasil 71,7% *versus* outros países 56,4%; p < 0,001). No Brasil, a percentagem de pacientes que receberam soluções cristaloides foi mais elevada (97,7% *versus* 76,8%; p < 0,001), e solução de cloreto de sódio a 0,9% foi o cristaloide mais comumente utilizado (62,5% *versus* 27,1%; p < 0,001). A análise multivariada sugeriu que os níveis de albumina se associaram com o uso tanto de cristaloides quanto de coloides, enquanto o tipo de prescritor dos fluidos se associou apenas com o uso de cristaloides.

**Conclusão:**

Nossos resultados sugerem que cristaloides são usados mais frequentemente do que coloides para ressuscitação no Brasil, e essa discrepância, em termos de frequências, é mais elevada do que em outros países. A solução de cloreto de sódio 0,9% foi o cristaloide mais frequentemente prescrito. Os níveis de albumina sérica e o tipo de prescritor de fluidos foram os fatores associados com a escolha de cristaloides ou coloides para a prescrição de fluidos.

## INTRODUCTION

Fluid resuscitation is defined as intravenous ﬂuid administration with the aim of improving tissue perfusion in shock states. It is one of the most common interventions in critically ill patients. Despite being a frequent intervention, fluid resuscitation lacks a clear definition. The choice of fluid to be administered as well as the dose and speed are not well determined, leading to differences in bedside practices.^(1,2)^

In the last 15 years, multiple randomized controlled trials and subsequent meta-analyses have shown that the type of fluid used for resuscitation, particularly hydroxyethyl starch (HES), may negatively affect outcomes.^(3-12)^ Even with recent published guidelines including new evidences,^(13,14)^ delays and failures with translating recommendations into practice are common, leading to variability in care.^(15,16)^ The Saline versus Albumin Fluid Evaluation - Translation of Research Into Practice Study (SAFE-TRIPS), a cross-sectional study conducted in 2007 including 391 intensive care units (ICUs) across 25 countries, reported that resuscitation practices varied significantly. Although colloid solutions were more expensive and may possibly be harmful in some patients, they were administered to more patients and during more resuscitation episodes than crystalloids.^(17)^

Recently, the same group conducted a similar observational study in a convenience sample of ICUs: the Fluid-TRIPS.^(18)^ This study demonstrated an important change in clinical practice, with a preferential use of crystalloids, specifically buffered salt solutions, over colloids. Another interesting finding of this study was that fluid choice was determined by local practice rather than by any identifiable patient characteristic.

The number of contributing ICUs from Brazil in the Fluid-TRIPS was just over half of all participating units, allowing the unique opportunity to separately analyze Brazilian data. Our hypothesis was that Brazilian ICUs would have different standards for fluid resuscitation, mainly regarding the choice of crystalloids.

Thus, the objective of this study was to describe current practices on fluid resuscitation in Brazilian ICUs and to compare Brazil with the other countries participating in the study.

## METHODS

This secondary analysis of a prospective, international, cross-sectional, observational study was carried out in a convenience sample of ICUs in 27 countries using the Fluid-TRIPS database, compiled in 2014.^(18)^

In Brazil, we recruited participating sites at critical care meetings through the Brazilian Research in Critical Care network (BRICNet) website and contacts and personal contacts with key opinion leaders. Participation was voluntary, and any hospital willing to join the study was considered eligible, with no exclusion criteria. The coordinating center was the *Universidade Federal de São Paulo,* and the institution’s Ethics and Research Committee approved the study protocol under the number CAAE 36093314.4.1001.5505 with a waiver for Informed Consent considering the observational nature of the study.

### Participants and data collection

In Brazil, the sites collected data on any single day between December 9^th^ and 11^th^ 2014. Methodological details were previously published.^(18)^ Briefly, the study day was defined as a 24-hour period. The investigators included all patients over 16 years old who required one or more fluid resuscitation episodes during the study period. There were no exclusion criteria. The total number of patients being treated in the ICUs on the study day was also recorded. We defined a fluid resuscitation episode as an hour during which a patient received a specifically prescribed intravenous fluid bolus of any crystalloid or colloid solution, a continuous infusion of 5mL/kg/hour or greater of crystalloid and/or any dose of colloid by continuous infusion.^(18)^

We recorded information on fluid availability in the participating ICUs as well as data related to patients, including demographic data, illness severity scores, admission diagnosis, laboratory test data, clinical data on the study day, predefined subgroup characteristics (trauma, traumatic brain injury - TBI, sepsis, and acute respiratory distress syndrome - ARDS), and information on the type and volume of fluids for resuscitation. The reason for fluid resuscitation and the prescriber characteristics were also recorded. We defined specialist or assistant physician as the board-certified intensivist or the physician responsible for the ICU on the study day. We defined senior resident or fellow as graduated students or residents in the last years of their residency, and we defined residents as those in the first years of their residency regardless of the specialty as it is usual in Brazil to have residents of different specialties in training.

We collected all data using an electronic data capture system (REDCap, Vanderbilt University, Tennessee, USA), hosted at *Instituto D’Or de Ensino e Pesquisa*, Rio de Janeiro, Brazil.

### Statistical analysis

Continuous variables are expressed as the mean ± standard deviation - SD or the median [interquartile range]. Categorical variables are expressed as counts (percentages). The comparison of the data between Brazil and other countries and between the administration of colloids and crystalloids in Brazilian patients were performed using a t-test or Wilcoxon rank-sum test for continuous data or Pearson’s chi-squared test for categorical data, as appropriate. Differences in the proportions of crystalloid and colloid episodes were tested using generalized estimating equations (GEEs), accounting for clustering at the patient level.

As in the main study,^(18)^ multivariable analyses using GEEs accounting for clustering at the patient level were conducted to determine associations between patient demographics, clinical characteristics and the type of fluid administered. We used 2 binary outcomes in the analysis: 1) crystalloid episode Yes *versus* crystalloid episode No, and 2) colloid episode Yes *versus* colloid episode No. The denominators of these two outcomes were the total number of fluid episodes; thus, as a given patient could have received crystalloids as well as colloids within the same hour (the same fluid episode), the total number of fluid episodes was higher than the sum of crystalloid episodes and colloid episodes. As these outcomes were analyzed separately, two different sets of odds ratios (ORs) were generated for each variable. Variables meeting a predetermined level of statistical significance (p < 0.1) with the administration of crystalloids or colloids in univariate models were included in the final multivariable model. Associations were considered statistically significant if p < 0.01. The results of the multivariable analysis are presented as adjusted ORs and 95% confidence intervals (95%CI). Details regarding the handling of missing data are provided in the main paper.^(18)^ All analyses were carried out using the R statistical software package, version 3.1.0 (2014-04-10).

## RESULTS

In Brazil, 217 ICUs participated in the study (participating centers are listed at the end of this manuscript). The overall summary of FLUID-TRIPS data is shown in table 1. Data on the participation of other countries can be found in detail in the main study.^(18)^ During the 24-hour study period, 3,214 patients were included in Brazil, of whom 519 (16.1%) received fluids. Almost half of the patients received fluids within the first two days of ICU admission (46%). The baseline characteristics of patients in Brazil and those of patients in the other countries are shown in table 2.

**Table 1:** Overall summary of Fluid-TRIPS

Variable	Brazil	Other countries	Total
Total number of participating ICU sites	217	209	426
Total number of ICU sites recruiting FLUID patients*	176	195	371
Total number of ICU patients	3,214	3,493	6,707
Total number of FLUID patients*	519	937	1,456
FLUID patients* among total ICU patients† (%)	16.1	26.8	21.7
Total number of fluid episodes	880	1,836	2,716

ICU - intensive care unit.

* FLUID patients: patients who required one or more fluid resuscitation episodes during the study period; †p < 0.001 for difference between Brazil and other countries (p-values of Pearson's Chi-squared test). Results expressed as n or %.

**Table 2:** Baseline characteristics of patients in Brazil and other countries

Variables	Brazil (n = 519)	Other countries (n = 937)	p value
Age (year)	63.0 (46.0 - 75.0)	64.0 (53.0 - 74.0)	0.061
Sex, male	296 (57.0)	582 (62.1)	0.058
APACHE II in 24 hours prior to survey day	18.0 (12.0 - 25.0)	18.0 (12.0 - 25.0)	0.910
Number of days in ICU	2.0 (1.0 - 6.0)	1.0 (0.0 - 7.0)	0.007
Patients receiving fluid resuscitation according to the number of days in the ICU at the study day			
Day 0	119/519 (22.9)	327/936 (34.9)	< 0.0001
Day 1	120/519 (23.1)	172/936 (18.4)	
Day 2	68/519 (13.1)	87/936 (9.3)	
Days 3 - 7	101/519 (19.5)	135/936 (14.4)	
Days 8 - 14	53/519 (10.2)	99/936 (10.6)	
Days 15 - 21	25/519 (4.8)	42/936 (4.5)	
Days 22 - 28	7/519 (1.3)	25/936 (2.7)	
Days 29 - 59	16/519 (3.1)	35/936 (3.7)	
Day ≥ 60	10/519 (1.9)	14/936 (1.5)	
Admission characteristics			
Operating room after elective surgery	137/519 (26.4)	243/936 (26.0)	0.185
Emergency room	132/519 (25.4)	198/936 (21.2)	
Hospital floor	83/519 (16.0)	169/936 (18.1)	
Operating room after emergency surgery	69/519 (13.3)	135/936 (14.4)	
Transferred from other ICU or hospital	49/519 (9.4)	117/936 (12.5)	
Hospital floor after previous ICU stay	49/519 (9.4)	74/936 (7.9)	
Admission diagnosis			
Nonsurgical	298/519 (57.4)	512/936 (54.7)	0.318
Surgical	221/519 (42.6)	424/936 (45.3)	
Trauma category at hospital admission			
No trauma	468/518 (90.3)	843/935 (90.2)	0.921
Trauma with TBI	14/518 (2.7)	23/935 (2.5)	
Trauma without TBI	36/518 (6.9)	69/935 (7.4)	
ARDS in 24 hours prior to survey day	32 (6.2)	83 (8.9)	0.070
Sepsis in 24 hours prior to survey day	205 (39.7)	345 (36.9)	0.293
APACHE II chronic health points criteria			
Chronic health points liver criteria	14/508 (2.8)	42/927 (4.5)	0.097
Chronic health points renal criteria	15/509 (2.9)	18/928 (1.9)	0.223
Chronic health points cardiac criteria	30/508 (5.9)	58/928 (6.2)	0.795
Chronic health points respiratory criteria	27/509 (5.3)	65/932 (7.0)	0.215
Chronic health points immunocompromised	66/511 (12.9)	91/929 (9.8)	0.069

APACHE - Acute Physiology and Chronic Health Evaluation; ICU - intensive care unit; TBI - traumatic brain injury; ARDS - acute respiratory distress syndrome. Summary statistics of continuous variables are presented as the median (interquartile range), with p-values based on the nonparametric test (i.e., Wilcoxon rank-sum test). Summary statistics of categorical variables are presented as percentages, with p-values based on Pearson's Chi-squared test.

In 880 fluid resuscitation episodes in Brazil, a specialist was the main fluid prescriber (82.3%), and the main indication for fluid resuscitation was impaired perfusion and/or low cardiac output (71.7%) (Table 3 and Table 1S in Supplementary material). The total volume of resuscitation fluid received and net fluid balance on the survey day were higher in Brazil than in other countries (Table 4).

**Table 3:** Indications for fluid resuscitation in Brazil and in other countries

Variables	Brazil	Other countries	p value‡
Indication for fluid in each fluid resuscitation episode	n = 877*	n = 1,820†	p < 0.0001
Impaired perfusion/low cardiac output	629 (71.7)	1,026 (56.4)	
Ongoing bleeding	25 (2.9)	38 (2.1)	
Other fluid losses	24 (2.7)	84 (4.6)	
Unit protocol	15 (1.7)	119 (6.5)	
Abnormal vital signs	175 (20.0)	518 (28.5)	
Other	9 (1.0)	35 (1.9)	
Fluid prescriber	n = 880	n = 1.836	p < 0.0001
Specialist/assistant physician	724 (82.3)	597 (32.5)	
Senior resident/fellow	92 (10.5)	706 (38.5)	
Resident	42 (4.8)	455 (24.8)	
Nurse	1 (0.1)	42 (2.3)	
Other	21 (2.4)	36 (2.0)	

Missing data: 0.3%; † missing data: 0.9%; ‡ generalized estimating equation model adjusted for the patient-level clustering effect.

**Table 4:** Characteristics of fluids received per patient in Brazil and other countries

Variable	Brazil (n = 519)	Other countries (n = 937)	p value
Patients received crystalloid	507 (97.7)	720 (76.8)	< 0.0001
Patients received colloid	38 (7.3)	356 (38.0)	< 0.0001
Total volume of resuscitation fluid received on survey day (mL)	1,000.0 (500.0 - 1,500.0)	550.0 (400.0 - 1,460.0)	< 0.0001
Total volume of crystalloid received on survey day (mL)	1,000.0 (500.0 - 1,500.0)	835.0 (500.0 - 1,500.0)	0.018
Total volume of colloid received on survey day (mL)	275.0 (100.0 - 500.0)	250.0 (100.0 - 500.0)	0.688
Total volume of fluid input on the survey day (mL)	3,059,0 (2,015.0 - 4,165.5)	3,343.0 (2,436.0 - 4,537.5)	< 0.0001
Total volume of fluid output on the survey day (mL)	1,385.0 (750.0 - 2,325.0)	2,050.0 (1,152.0 - 3,310.0)	< 0.0001
Net fluid balance on the survey day (mL)	1,310.0 (500.0 - 2,517.0)	1,018.0 (150.0 - 2,350.0)	0.002

Summary statistics of continuous variables are presented as the median (interquartile range), with p-values based on the nonparametric

**Figure 1:**
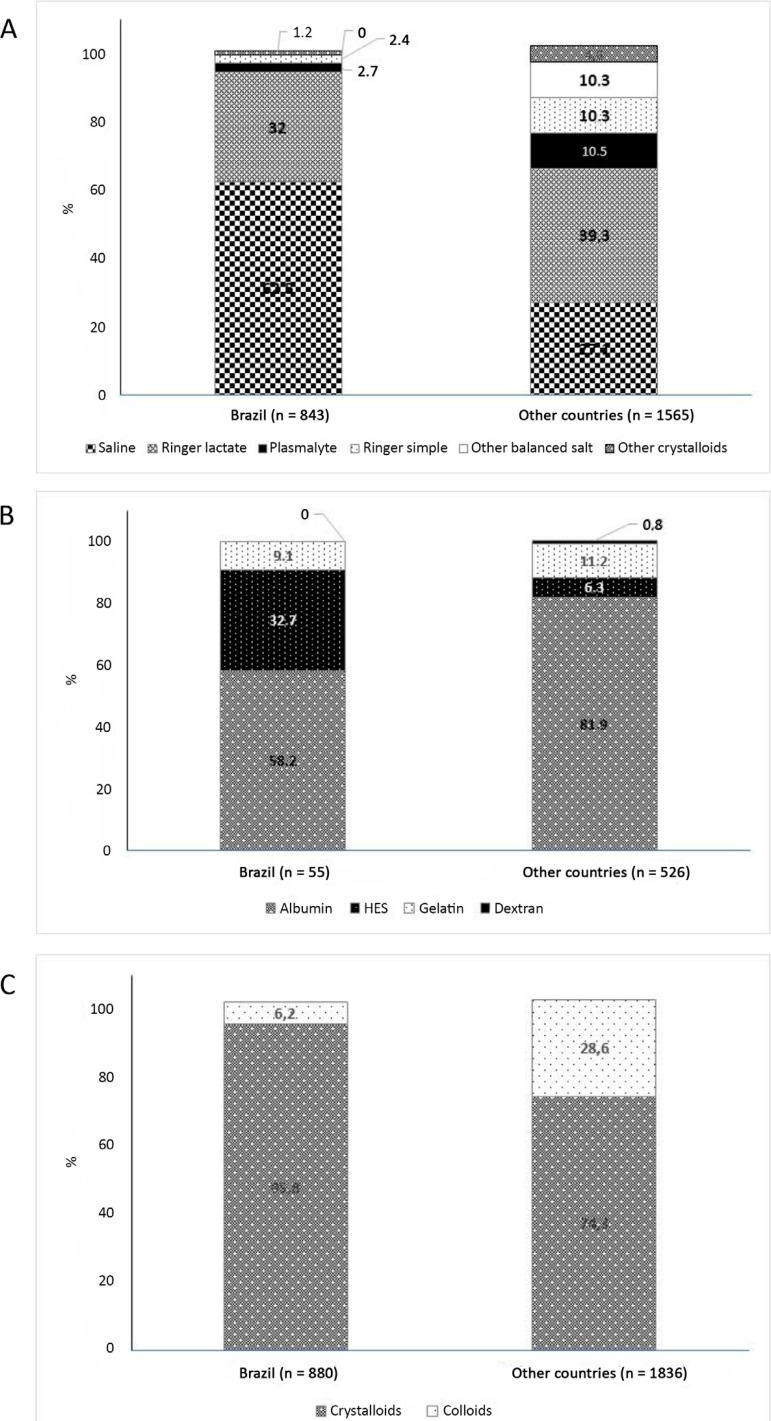
Percentage of fluid resuscitation episodes in Brazil and other countries. (A) Comparison of the choice of fluid in each of the fluid episodes. (B) Comparison of the choice of crystalloids in episodes in which crystalloids were used. (C) Comparison of the choice of colloids in episodes in which colloids were used. Percentages may not add to 100%, as patients can be administered more than one type of fluid during resuscitation episodes. HES - hydroxyethyl starch.

Compared to other countries, crystalloid solutions were more frequently used than colloid solutions in Brazil (Figure 1). In Brazil, 0.9% sodium chloride was significantly more commonly used than in other countries (62.5% *versus* 27.1%, p < 0.0001) (Table 1S - Supplementary material), despite the availability of different fluids at the participating ICUs (Table 2S - Supplementary material). In Brazil and other countries, the most commonly used balanced crystalloid solution was Ringer’s lactate. Plasma Lyte was used more frequently in other countries than in Brazil (Table 1S - Supplementary material). The percentage of patients receiving crystalloid or colloid solutions or the number of crystalloid or colloid episodes were not modified in the presence of trauma, TBI, sepsis or ARDS. These conditions did not lead to significant changes in the total volume of resuscitation fluid received on the survey day. However, patients with sepsis and ARDS had a higher net fluid balance on the survey day (Table 3S to Table 6S - Supplementary material).

We analyzed the factors associated with the choice of crystalloids or colloids for fluid resuscitation episodes. The multivariable analysis (Table 5) suggested that, in Brazil, lower albumin levels (i.e., < 27g/dL, ≥ 27g/dL, or missing), in general, were associated with both the use of crystalloids and colloids (p = 0.001 and < 0.0001, respectively).

**Table 5:** Multivariate analysis of factors associated with the choice of crystalloid or colloid for fluid resuscitation episodes in Brazilian patients

Variable	Crystalloid given OR (95%CI)	p value	Colloid given OR (95%CI)	p value
Admission characteristics				
Operating room after elective surgery	1.0	0.215	1.0	0.1144
Emergency room	1.0 (0.3 - 2.9)		0.6 (0.2 - 1.5)	
Hospital floor	3.0 (0.3 - 26.8)		0.2 (0.0 - 1.2)	
Transferred from other ICU or hospital	2.5 (0.3 - 22.6)		0.7 (0.2 - 3.1)	
Operating room after emergency surgery	0.6 (0.1 - 2.6)		0.8 (0.2 - 2.7)	
Hospital floor after previous ICU stay	0.3 (0.1 - 1.1)		2.4 (0.8 - 7.5)	
Fluid prescriber				
Specialist/assistant physician	1.0	< 0.0001	1.0	0.1483
Senior resident/fellow	9.9 (3.6 - 27.7)		0.2 (0.0 - 1.1)	
Resident	0.6 (0.1 - 3.9)		1.4 (0.3 - 6.5)	
Metabolic acidosis				
No	1.0	0.241	1.0	0.2207
Yes	0.5 (0.1 - 1.8)		1.3 (0.5 - 3.4)	
Missing	0.3 (0.1 - 1.2)		2.5 (0.9 - 7.1)	
Lactate (mmol/L) categories				
< 2	1.0	0.394	1.0	0.8014
≥ 2	0.9 (0.2 - 3.5)		0.8 (0.3 - 2.1)	
Missing	0.4 (0.1 - 1.8)		1.1 (0.4 - 3.0)	
Mean arterial pressure (per 10mmHg decrease)	1.2 (1.0 - 1.5)	0.012	0.9 (0.7 - 1.0)	0.0669
Albumin (g/L) categories				
< 27	1.0	0.001	1.0	< 0.0001
≥ 27	8.6 (0.8 - 89.8)		0.2 (0.0 - 0.9)	
Missing	7.2 (2.5 - 20.7)		0.2 (0.1 - 0.4)	

OR - odds ratio; 95%CI - 95% confidence interval; ICU - intensive care unit. The results were generated from a generalized estimating equation model with patient ID as a cluster, using two binary outcomes in the analysis: (1) crystalloid episode Yes versus crystalloid episode No, and (2) colloid episode Yes versus colloid episode No. The denominators of these two outcomes were the total number of fluid episodes; thus, a given patient could have received a crystalloid as well as a colloid within the same hour (the same fluid episode). The analysis included 844 episodes from 503 study participants, as data were lost due to missing values that could not be included in the multivariate analysis. This number represents a loss of 4.1% of episodes and 3.1% of study participants.

Among the patients who received crystalloids, the odds of having an albumin level ≥ 2g/dL were 9.4 times (OR = 8.6 [0.8 - 89.8]) that of having an albumin level < 27g/dL.

There was also a higher chance of having unknown/missing values for albumin (OR = 7.2, 95%CI = 2.5 - 20.7) than having an albumin level < 27g/dL. Similarly, among those who received colloids, the odds of having an albumin level ≥ 27g/dL was one-fiftieth (OR = 0.2 [0.0 - 0.9]) that of having levels < 27g/dL. In addition, for patients receiving crystalloids, the odds of them being prescribed by a senior resident/fellow was 9.9 times higher (OR = 9.9, 95%CI = 3.6 - 27.7) than that of them being prescribed by a specialist/assistant physician. For patients receiving colloids, there was no clear association with fluid prescriber. The univariate analysis is available in table 7S (Supplementary material).

## DISCUSSION

Our results demonstrated that in Brazil, crystalloids were more frequently used than colloids for fluid resuscitation. In other countries, crystalloids were also the fluid of choice, but in Brazil, the proportion was significantly higher. Sodium chloride (0.9%) was the most prescribed crystalloid in Brazil, despite the availability of balanced solutions. In other countries, balanced solutions were the preferred crystalloids. The availability of serum levels and the current albumin level were the factors associated with the choice of crystalloids or colloids for fluid resuscitation. In addition, the type of fluid prescriber was significantly associated with crystalloid use.

The results in Brazil are consistent with more recent studies regarding fluid resuscitation practices. Fluid resuscitation aims at improving tissue perfusion by restoring the perfusion pressure of vital organs and ensuring adequate cardiac output.^(13)^ Aligned with these principles, the main indications for fluid administration in Brazilian ICUs were similar to those found in the main study and in other studies addressing this issue.^(18,19)^ Our results also showed a reduction in the use of colloid solutions.^(18-20)^ The evidence of harm from recent randomized clinical trials (RCTs) with synthetic colloids such as HES (3-12) could explain the preference for crystalloid solutions in Brazil and in other countries. It is interesting to note that the higher proportion of the use of colloids in other countries is represented by the use of albumin. As albumin is expensive, the costs may have limited its use in Brazil, a middle-income country.^(21)^

Another aspect that differentiates Brazil from other countries was the use of 0.9% sodium chloride as the crystalloid solution of choice. Although Plasmalyte is a high-cost balanced solution in Brazil, there are low-cost balanced solutions available (e.g., Ringer’s lactate). Our study was not designed to assess the potential reasons for this difference between Brazil and other countries. It is possible that this was influenced by the variation in availability among the sites and countries, which would bias any further analysis. The relatively small number of patients and variables in our database might also compromise the reliability of eventual findings. Another possible explanation is a cultural preference derived from years of using saline potentially associated with a reduced awareness of the potential adverse effects of hyperchloremic solutions, as the controversy around balanced vs. unbalanced crystalloids was not as intense as it is currently.^(22-24)^ We believe our findings are potentially useful for hypothesis generation, and further studies are necessary to better evaluate potential factors associated with this choice.

Sepsis, ARDS, trauma and TBI did not influence the choice between colloids and crystalloids. The uncertainty about the ideal fluid for these specific diseases could explain this finding.^(25)^ However, in Brazilian ICUs, albumin serum levels had a clear role in guiding the choice of fluid. This preference is not supported by the available evidence. The results from high-quality RCTs suggest that intravenous albumin administration does not reduce the mortality rate in mixed populations of critically ill patients, including those who have hypoalbuminemia.^(26)^ Even albumin supplementation in addition to crystalloids targeting serum concentrations higher than 30g per liter in septic patients did not improve survival at 28 and 90 days.^(27)^ Thus, we believe that this finding probably reflects local practice patterns rather than solid evidence. It is worth mentioning that senior residents and fellows were more likely to prescribe crystalloid fluids to patients than specialists, probably suggesting that academic exposure to scientific evidence promotes changes in practice behaviors.^(28)^ Another potential explanation is the generation difference. The specialists were previously exposed to a cultural environment in which colloids were heavily used based on their potential better effect on oncotic pressure. In contrast, the new generation, composed of residents and fellows, was exposed to scientific evidence of harm with colloid use. This also suggests that continuous training, even for specialists, is important to ensure better quality of care.

This study has strengths and some limitations, some of which were mentioned in the main study.^(18)^ This is the first study to describe resuscitation fluid practices in a large sample of Brazilian ICUs. The use of standard case report forms and definitions across all countries and detailed information on clinical factors that may potentially influence the choice of fluid for resuscitation at the time of the fluid episode allowed not only comparisons with other countries but also analyses of national practice patterns. Among the limitations of the study, it is important to mention the generalizability of the results. Even with a large sample of ICUs, the use of convenience sampling might have not reflected practices adopted in all Brazilian ICUs. Another limitation is the definition of fluid resuscitation episodes.^(18)^ Finally, the interpretation of fluid administration practices in specific patient populations, such as those with sepsis, requires caution due to relatively small patient numbers.

## CONCLUSION

Crystalloids were more frequently used than colloids for fluid resuscitation in Brazilian intensive care units. Sodium chloride (0.9%) was the most prescribed crystalloid in Brazil, despite the availability of balanced solutions. The availability of serum levels and the low albumin level were the factors that influenced the choice between crystalloid or colloid for fluid resuscitation. In addition, senior residents/fellows were more likely to prescribe crystalloid fluids to patients than specialists.

## Appendix

Brazilian participating sites Country coordinator: Flavia R Machado. Albert Sabin Hospital e Maternidade - SR Zajac; Associação Beneficente Hospital Unimar - A Campos, D de Albuquerque; Associação Hospitalar Beneficente São Vicente de Paulo - J Gomez; Casa de Caridade de Carangola - S Vaz; Casa de Saúde Campinas - B Campos, W Delgadinho; Casa de Saúde Santa Lúcia - RT Amâncio, VC Souza-Dantas; Clínica Campo Grande - V Damasceno, J dos Santos; Clínica Dom Rodrigo - F de Araújo, I do Nascimento; Complexo Hospitalar Ortotrauma de Mangabeira - F de Araújo, I do Nascimento; Fundação Doutor Amaral Carvalho - M Higashi, E Mattos; Fundação Pio XII- Hospital de Câncer de Barretos - CP Amendola, UVA Silva; Hospital São José - F Dal-Pizzol, C Ritter; Hospital 9 de Julho - UTI 10a andar - MD D’Agostino; Hospital 9 de Julho - UTI 11a andar - C Moreira; Hospital 9 de Julho - UTI 1a andar - C Moreira; Hospital 9 de Julho - UTI 8a andar - L da Cruz Neto; Hospital 9 de Julho - UTI 9a andar - F Ganem; Hospital Adventista de Belém - ME de Oliveira, E Sobrinho; Hospital Adventista de Manaus - P Ferreira, R Rabelo; Hospital Alemão Oswaldo Cruz - R Cordioli, F Zampieri; Hospital Alvorada Brasília - ACC Cembranel, EJ Nascimento; Hospital Alvorada Taguatinga - RS Biondi, E Milhomem; Hospital Amecor - Unidade Coronariana - M Bley; Hospital Amecor - UTI Geral - M Bley; Hospital Anis Rassi - G Canedo, R Filho; Hospital Assunção - M Fukushima, L Miilher; Hospital Beneficência Portuguesa - UTI do Choque - S Houly; Hospital Brigadeiro - EC Maitan, OL Santarém; Hospital Carlos da Silva Lacaz - A Ferreira, E Ferreira; Hospital Casa de Saúde de Santos - P Rosateli, A Scazufka; Grupo Hospitalar Nossa Senhora da Conceição - W Nedel, VM Oliveira; Hospital Copa D’Or - CTI Amarelo - L Rabello, W Viana; Hospital Copa D’Or UPO 2 - AP Santos, W Viana; Hospital Copa D’Or - UTI Azul - L Tanaka, W Viana; Hospital Copa D’Or - UTI Pós-Operatória - L Salles, AP Santos; Hospital Copa D’Or - CTI Verde - K Ebecken, W Viana; Hospital Copa D’Or - Neurointensiva - D Musse, L Rabello; Hospital Copa D’Or - UTI Lilás - L Rabello, L Tanaka; Hospital da Luz Vila Mariana - F Filho, F dos Santos Borges; Hospital da Restauração - K Monteiro, F Buarque; Hospital das Clínicas da Faculdade de Medicina da Universidade de São Paulo - UTI Emergências Clínicas - P Mendes, L Taniguchi; Hospital das Clínicas da Faculdade de Medicina de Botucatu - L de Stefano, A Gut; Hospital das Clínicas da Faculdade Ribeirão Preto - M Auxiliadora-Martins, ML Puga; Hospital das Clínicas da Universidade Federal de Minas Gerais - V Nobre; Hospital das Clínicas da Universidade Federal do Espírito Santo - LM Caixeta, PF Vassallo; Hospital das Clínicas de Porto Alegre - RB Moraes, J Vidart; Hospital de Base - Faculdade de Medicina de São José do Rio Preto - H Batista, SM Lobo; Hospital de Caridade Astrogildo de Azevedo - CB da Silva, C Kmohan; Hospital de Clínicas Gaspar Vianna - C da Rocha, H Reis; Hospital de Urgência - UTI Geral 1 - D Pedroso, J Sobrinho; Hospital de Urgência - UTI Geral 4 - S Faria; Hospital de Urgência - UTI Neurológica 3 - J Sobrinho; Hospital de Urgência - UTI Trauma 2 - S Faria, D Pedroso; Hospital Distrital Evandro Ayres de Moura - L Figueiredo, H Magalhaes; Hospital do Coração - MLP Romano, R Vasconcelos; Hospital do Coração do Brasil - H Araújo, M de Araújo; Hospital do Rim e Hipertensão - AT Bafi, FGR Freitas; Hospital do Servidor Público Estadual - S Luzzi, D Ortega; Hospital do Servidor Público Municipal de São Paulo - T Farhat; Hospital do Servidor Público Municipal de São Paulo - UTI 7a andar - KM Sato; Hospital do Subúrbio - J Motta, C Lins; Hospital do Trabalhador - A Rea-Neto, F Reese; Hospital Dom Hélder - RAF Gomes, ARA Macedo Júnior; Hospital Dom Vicente Scherer - EM Rodrigues Filho, M Hadrich; Hospital e Maternidade Municipal Dr. Odelmo Leão Carneiro - C Arantes, MAS Toneto; Hospital e Maternidade Otaviano Neves - B Fernandino, A Pereira; Hospital e Pronto-Socorro 28 de Agosto - L Cavalcante, A Matos; Hospital Ecoville - L Araújo, A Rea-Neto; Hospital Escola da Faculdade de Medicina de Jundiaí - E Ferreira; Hospital Estadual de Urgência e Emergência de Vitória - L Dornelas, L Tcherniacovsk; Hospital Estadual Getúlio Vargas - UTI 1 - A Rodrigues, K Schechter; Hospital Estadual Getúlio Vargas - UTI 2 - F Montesanto, B Vidal; Hospital Estadual Getúlio Vargas - UTI 3 - C Frambach, G Moralez; Hospital Estadual Getúlio Vargas - UTI 4 - F Callil, V Montez; Hospital Estadual Rocha Faria - CHF Ramos; Hospital Evangélico de Londrina - J Festti, C Grion; Hospital Evangélico de Sorocaba - A de Souza, M Marabezi; Hospital Federal dos Servidores do Estado RJ - M Bissoli, J Marques; Hospital Felício Rocho - D Fontes, C Ranyere; Hospital Fernandes Távora - A Batista, L Martins; Hospital e Maternidade Galileo - W Delgadinho, M Rocha; Hospital Geral de Fortaleza - UTI Azul - C Feijó, V Araújo; Hospital Geral de Goiânia - D Pedroso, G Silva; Hospital Geral de Vitória da Conquista - M Martins, M Ribeiro II; Hospital Geral Dr. César Cals - A Justo, A Macedo; Hospital Goiânia Leste - M Nobrega, M Nobrega; Hospital Hélio Anjos Ortiz - H Júnior, M Lazzarotto; Hospital IBR - J Andrade, L Souza; Hospital Estadual Ipiranga - S Fernandes, F Lombardi; Hospital Israelita Albert Einstein - TD Correa, M Assunção; Hospital Jardim Amália - C Arbex, M Arbex; Hospital Estadual Jayme Santos Neves - F dos Anjos Sad, E Stucchi; Hospital M’Boi Mirim - A Andrade, C de Abreu Filho; Hospital Madre Regina Prottman - D Colodetti, M Rodrigues; Hospital Marcelino Champagnat - M de Oliveira, A Rea-Neto; Hospital de Clínicas Mário Lioni - P Galhardo, A Japiassú; Hospital Maternidade e Pronto-Socorro Santa Lucia - R Bergo, F Dall’Orto; Hospital Maternidade São José - P Bernardes, R Figueiredo; Hospital Memorial São José - G Costa, K Monteiro; Hospital Moinhos de Vento - M Rosa, JHD Barth; Hospital Municipal de Paracatu - T Neiva, R de Souza; Hospital Municipal Dr. Munir Rafful - M Arbex, L de Oliveira; Hospital Municipal Irmã Dulce - D Boni, MOG Douglas; Hospital Municipal Dr. José Soares Hungria - K Conde, N Quintino; Hospital Municipal Padre Germano Lauck - R Almeida, J Fuck; Hospital Municipal Pedro II - E Paranhos, J Soares; Hospital Municipal Santa Isabel - A de Carvalho, C Tavares; Hospital Municipal São José - D Possamai, G Westphal; Hospital Nereu Ramos - E Berbigier, I Maia; Hospital Norte D’Or - J Pinto, S Sant’Anna; Hospital Nossa Senhora da Conceição - JM de Araújo, F Schuelter-Trevisol; Hospital Nossa Senhora dos Prazeres - A Gargioni, R Gargioni; Hospital Nossa Senhora Monte Serrat - MAP Alves; Hospital Novo Atibaia - A Bemfica, R Franco; Hospital Ortopédico - L da Silva, M Nobrega; Hospital Paulistano - I Campos, DT Noritomi; Hospital Paulo Sacramento - ELA Ferreira; Hospital PIO XII de São José dos Campos - Unidade Coronariana - M Durval, A Silva; Hospital Português - R Hermes, O Messeder; Hospital Primavera - J Feijó, E Nogueira; Hospital Professor Edmundo Vasconcelos - E Jodar, R Pereira; Hospital Regional de Sousa - P da Silveira, A Lunguinho; Hospital Regional de Itapetininga São Camilo - V Irineu, R Seabra; Hospital Regional de Jundiaí - G Cavalcanti, M Leão; Hospital Regional de Presidente Prudente - GN Betônico, LA Garcia; Hospital Regional de Samambaia - UTI 1 - F Amorim, C de Carvalho; Hospital Regional de Samambaia - UTI 2 - S Margalho, F Santos; Hospital Renascentista - D Beraldo, R dos Santos; Hospital Samaritano Rio de Janeiro - J Freitas, R Lima; Hospital Samaritano São Paulo - UTI 6a andar - B Mazza, S Almeida; Hospital Samaritano São Paulo - 3a andar - B Mazza, R Rocha; Hospital Samaritano João Pessoa - P Gottardo, C Mendes; Hospital Santa Helena - R Narciso, S Pantaleão; Hospital Santa Isabel - K Gerent; Hospital Santa Izabel - R Marco, D Vinho; Hospital Santa Juliana - EMV Troncoso, KLN Vilassante; Hospital Santa Lúcia - A Ventura, M da Silva; Hospital Santa Maria - M Nobrega, F Oliveira; Hospital Santa Maria - Intensibarra - I Santiago, A Lima; Hospital Santa Rita - F da Costa, M Vilela; Hospital Santa Rita - T Lisboa, A Torelly; Hospital São Camilo Ipiranga - M Dutra, F Giannini; Hospital São Camilo Pompéia - A Ramacciot, AT Maciel; Hospital São Francisco de Assis - GA da Silva, M da Silva; Hospital São João de Deus - G Gussen, M Rocha; Hospital São Lucas - UTI cirúrgica - C Santos, T Smith; Hospital São Lucas - UTI clínica - A Sobrinho, T Smith; Hospital São Lucas da Pontifícia Universidade Católica do Rio Grande do Sul - S Baldiserotto, M Moretti; Hospital São Marcos - UTI A - W Dantas, L Ishiy; Hospital São Marcos - UTI B - W Dantas, L Ishiy; Hospital São Mateus - JG Moreira Filho; Hospital Saúde da Mulher - N Machado, L Rezegue; Hospital Sepaco - AT Bafi, ES Pacheco; Hospital SOS Cárdio - F Aranha, R Saorin; Hospital Tereza Ramos - K de Paula, R Waltrick; Hospital TotalCor - A Batista, P de Barros e Silva; Hospital Uniclinic - M Serpa, J Terceiro; Hospital Unimed ABC - MOG Douglas, R Rosenblat; Hospital Unimed de Belo Horizonte - A Barbosa, C Nogueira; Hospital Unimed de Limeira - A de Carvalho, L Paciência; Hospital Unimed de Macaé - JT Passos, PTS Almeida; Hospital Unimed de Manaus - WO Filho, MM Lippi; Hospital Unimed Rio de Janeiro - M Assad, F Miranda; Hospital Unimed Rio de Janeiro - UTI Cardio - R Gomes, P Nogueira; Hospital Unimed Salto - MAP Alves; Hospital Universitário Cajuru - V Bernardes, L Tannous; Hospital Universitário Ciências Médicas - R Dutra, G Mirachi; Hospital Universitário da Universidade Federal de Juiz de Fora - BV Pinheiro, EV Carvalho; Hospital Universitário da Universidade Federal de São Paulo - UTI Clínica Médica - H Guimaraes, L Vendrame; Hospital Universitário da Universidade Federal de São Paulo - UTI Geral - F Machado, A Nascente; Hospital Universitário da Universidade Federal de São Paulo - UTI Neuro - F Machado, J Polezei; Hospital Universitário da Universidade Federal de São Paulo - UTI Pronto-Socorro - AFT de Góis, KMC Teixeira; Hospital Universitário da Faculdade de Medicina de Jundiaí - G Cavalcanti, M Leão; Hospital Universitário de Maringá - A Germano, S Yamada; Hospital Universitário de Santa Cruz do Sul - P de Moraes, R Foernges; Hospital Universitário de Santa Maria - L Garcia, S Ribeiro; Hospital Universitário Getúlio Vargas - WO Filho, A Matos; Hospital Universitário Júlio Müller - D Castiglioni, G da Silva; Hospital Universitário Lauro Wanderley - P Gottardo, C Mendes; Hospital Universitário Maria Aparecida Pedrossian - S Pinto; Hospital Universitário São Francisco de Paula - M Guerreiro, L Teixeira; Hospital Universitário -Universidade Federal Grande Dourados - M Matsui, E Neto; Hospital Vila da Serra - F Anselmo, H Urbano; Hospital Vita Batel - R Deucher, A Rea-Neto; Instituto do Coração, Hospital das Clínicas da Faculdade de Medicina da Universidade de São Paulo - J Ferreira, E Costa; Instituto do Coração, Hospital das Clínicas da Faculdade de Medicina da Universidade de São Paulo - REC - FRBG Galas, LA Hajjar; Instituto do Coração, Hospital das Clínicas da Faculdade de Medicina da Universidade de São Paulo - FG Lima, VRB Benites; Instituto de Infectologia Emílio Ribas II - R Borba, M Douglas; Instituto de Ortopedia e Traumatologia - CPP Castro, AB Saraiva; Instituto de Pesquisa Clínica Evandro Chagas - Instituto de Pesquisa Clínica Evandro Chagas da Fundação Oswaldo Cruz - FA Bozza, A Japiassú; Instituto do Câncer do Estado de São Paulo - JP Almeida, LA Hajjar; Instituto Estadual do Cérebro Paulo Niemeyer - C Righy, B Goncalves; Instituto D’Or de Ensino e Pesquisa - G Viana, A Reis; Instituto Latino Americano de Sepse - F Carrara, A Carvalho Júnior; Instituto Nacional de Cardiologia - M de Freitas, R Felipe; Instituto Ortopédico - L Caetano, M Nobrega; Instituto de Pesquisa Hospital do Coração - D de Moraes Paisani; Irmandade de Misericórdia de Guaxupé - SA Bezerra, DRB Pereira; Irmandade Misericórdia Hospital Santa Casa de Monte Alto - L Cassimiro, W Filho; Lifecenter - M Hermeto, B Pinto; Samur - L Ferraz, L Melo; Santa Casa de Angra dos Reis - V Bogado, S Silva; Santa Casa de Belém do Pará - R Batista, N Fonseca; Santa Casa de Belo Horizonte - P Correia, G Reis; Santa Casa de Caridade de Diamantina - MF Sousa, MMF Souza; Santa Casa de Misericórdia de Assis - GN Betônico, AL Leonardi; Santa Casa de Caridade de Don Pedrito - J Alvarez, A Tarouco; Santa Casa de Misericórdia de Paraguaçu Paulista - JA Alves, PRG Silva; Santa Casa de Misericórdia de Porto Alegre - G Friedman, T Lisboa; Santa Casa de Misericórdia de Presidente Prudente - C Bosso, G Plantier; Santa Casa de Misericórdia de Ribeirão Preto - P Antoniazzi, F Ostini; Santa Casa de Misericórdia de Santana do Livramento - J Alvarez, D de Souza; Santa Casa de Misericórdia de Santo Amaro - P Chaves, J Farhat Júnior; Santa Casa de Misericórdia de São Paulo - R Marco, E Peixoto; Santa Casa de Misericórdia de Vitória da Conquista - G Moreno; Santa Casa Maringá - Universidade Estadual Maringá - D Bolognese, P Torres; São Bernardo Apart Hospital - R López, M Rodrigues; Sociedade Beneficente de Senhoras Hospital Sírio-Libanês - LCP Azevedo, F Ramos; Universidade Estadual de Campinas - UTI da Disciplina de Emergências Clínicas - C Gontijo-Coutinho, T Santos; Universidade Estadual de Londrina - C Grion, M Tanita; Vitória Apart Hospital - A Muniz, C Piras The other countries sites are mentioned in the original publication.
