# Supplementary material for: Resuscitation fluid practices in Brazilian intensive care units: a secondary analysis of Fluid-TRIPS
Source: Rev Bras Ter Intensiva. 2021 Apr-Jun;33(2):206–18. doi: 10.5935/0103-507X.20210028 (PMC8275089; doi:10.5935/0103-507X.20210028)
Supplement: Supplementary file 1 [file rbti-33-02-0206-suppl01.pdf]

Resuscitation fluid practices in Brazilian intensive care units: a secondary analysis of Fluid-TRIPS

Práticas de ressuscitação volêmica em unidades de terapia intensiva brasileiras: uma análise secundária do estudo Fluid-TRIPS

Flavio Geraldo Rezende de Freitas[1], Naomi Hammond[2], Yang Li[2], Luciano Cesar Pontes de Azevedo[3,4], Alexandre Biasi Cavalcanti[5], Leandro Taniguchi[4], André Gobatto[4], André Miguel Japiassú[6,7], Antonio Tonete Bafi[1,8], Bruno Franco Mazza[9], Danilo Teixeira Noritomi[10], Felipe Dal-Pizzol[11], Fernando Bozza[7,12], Jorge Ibrahin Figueira Salluh[12], Glauco Adrieno Westphal[13], Márcio Soares[12], Murillo Santucci César de Assunção[14], Thiago Lisboa[15], Suzana Margarete Ajeje Lobo[16], Achilles Rohlfs Barbosa[17], Adriana Fonseca Ventura[18], Ailson Faria de Souza[19], Alexandre Francisco Silva[20], Alexandre Toledo[21], Aline Reis[12], Allan Cembranel[22], Alvaro Rea Neto[23,24,25], Ana Lúcia Gut[26], Ana Patricia Pierre Justo[27], Ana Paula Santos[28], André Campos D. de Albuquerque[29], André Scazufka[30], Antonio Babo Rodrigues[31], Bruno Bonaccorsi Fernandino[32], Bruno Goncalves Silva[33], Bruno Sarno Vidal[31], Bruno Valle Pinheiro[34], Bruno Vilela Costa Pinto[35], Carlos Augusto Ramos Feijo[36], Carlos de Abreu Filho[37], Carlos Eduardo da Costa Nunes Bosso[38] Carlos Eduardo Nassif Moreira[39], Carlos Henrique Ferreira Ramos[40], Carmen Tavares[41], Cidamaiá Arantes[42], Cintia Grion[43,44], Ciro Leite Mendes[45], Claudio Kmohan[46], Claudio Piras[47], Cristine Pilati Pileggi Castro[48], Cyntia Lins[49], Daniel Beraldo[50], Daniel Fontes[51], Daniela Boni[52], Débora Castiglioni[53], Denise de Moraes Paisani[5], Durval Ferreira Fonseca Pedroso[54,55], Ederson Roberto Mattos[56], Edgar de Brito Sobrinho[57], Edgar M. V. Troncoso[58], Edison Moraes Rodrigues Filho[59], Eduardo Enrico Ferrari Nogueira[60], Eduardo Leme Ferreira[61,62], Eduardo Souza Pacheco[1,63], Euzebio Jodar[64], Evandro L. A. Ferreira[65], Fabiana Fernandes de Araujo[66,67], Fabiana Schuelter Trevisol[68], Fábio Ferreira Amorim[69], Fabio Poianas Giannini[70], Fabrício Primitivo Matos Santos[69], Fátima Buarque[71], Felipe Gallego Lima[72], Fernando Antonio Alvares da Costa[73], Fernando Cesar dos Anjos Sad[74], Fernando G. Aranha[75], Fernando Ganem[39], Flavio Callil[31], Francisco Flávio Costa Filho[76], Frederico Toledo Campo Dall´Arto[77], Geovani Moreno[78], Gilberto Friedman[79], Giulliana Martines Moralez[31], Guilherme Abdalla da Silva[80], Guilherme Costa[81], Guilherme Silva Cavalcanti[82], Guilherme Silva Cavalcanti[83], Gustavo Navarro Betônico[84], Gustavo Navarro Betônico[85], Hélder Reis[86], Helia Beatriz N. Araujo[87], Helio Anjos Hortiz Júnior[88], Helio Penna Guimaraes[1], Hugo Urbano[89], Israel Maia[90], Ivan Lopes Santiago Filho[91], Jamil Farhat Júnior[92], Janu Rangel Alvarez[93,94], Joel Tavares Passos[95], Jorge Eduardo da Rocha Paranhos[96], José Aurelio Marques[97], José Gonçalves Moreira Filho[98], Jose Neto Andrade[99], José Onofre de C Sobrinho[54], Jose Terceiro de Paiva Bezerra[100], Juliana Apolônio Alves[101], Juliana Ferreira[4], Jussara Gomes[102], Karina Midori Sato[103], Karine Gerent[104], Kathia Margarida Costa Teixeira[1], Katia Aparecida Pessoa Conde[105], Laércia Ferreira Martins[106], Lanese Figueirêdo[107], Leila Rezegue[108], Leonardo Tcherniacovsk[109], Leone Oliveira Ferraz[110], Liane Cavalcante[111], Ligia Rabelo[28], Lilian Miilher[112], Lisiane Garcia[113], Luana Tannous[114], Ludhmila Abrahão Hajjar[72,115], Luís Eduardo Miranda Paciência[116], Luiz Monteiro da Cruz Neto[39], Macia Valeria Bley[117], Marcelo Ferreira Sousa[118], Marcelo Lourencini Puga[119], Marcelo Luz Pereira Romano[120], Marciano Nobrega[121,122,123], Marcio Arbex[124,125], Márcio Leite Rodrigues[126], Márcio Osório Guerreiro[127], Marcone Rocha[128], Maria Angela Pangoni Alves[129], Maria Angela Pangoni Alves[130], Maria Doroti Rosa[131], Mariza D'Agostino Dias[39], Miquéias Martins[132],Mirella de Oliveira[133], Miriane Melo Silveira Moretti[134], Mirna Matsui[135], Octavio Messender[136], Orlando Luís de Andrade Santarém[137], Patricio Júnior Henrique da Silveira[138], Paula Frizera Vassallo[139], Paulo Antoniazzi[140], Paulo César Gottardo[141], Paulo Correia[142], Paulo Ferreira[143], Paulo Torres[144], Pedro Gabrile M. de Barros e Silva[145], Rafael Foernges[146], Rafael Gomes[147], Rafael Moraes[148], Raimundo Nonato filho[149], Renato Luis Borba[150], Renato V Gomes[151], Ricardo Cordioli[152], Ricardo Lima[153], Ricardo Pérez López[154], Ricardo Rath de Oliveira Gargioni[155], Richard Rosenblat[156], Roberta Machado de Souza[157], Roberto Almeida[158], Roberto Camargo Narciso[159], Roberto Marco[160,161], Roberto waltrick[162], Rodrigo Biondi[163], Rodrigo Figueiredo[164], Rodrigo Santana Dutra[165], Roseane Batista[166], Rouge Felipe[167], Rubens Sergio da Silva Franco[168], Sandra Houly[169], Sara Socorro Faria[54], Sergio Felix Pinto[170], Sergio Luzzi[171], Sergio Sant'ana[172], Sergio Sonego Fernandes[173], Sérgio Yamada[174], Sérgio Zajac[175], Sidiner Mesquita Vaz[176], Silvia Aparecida Bezerra Bezerra[177], Tatiana Bueno Tardivo Farhat[103], Thiago Martins Santos[178], Tiago Smith[179], Ulysses V. A. Silva[180], Valnei Bento Damasceno[181], Vandack Nobre[182], Vicente Cés de Souza Dantas[183], Vivian Menezes Irineu[184], Viviane Bogado[185], Wagner Nedel[186], Walther Campos Filho[187], Weidson Dantas[188], William Viana[28], Wilson de Oliveira Filho[189,190], Wilson Martins Delgadinho[191,192], Simon Finfer[2], Flavia Ribeiro Machado[1], on behalf of Fluidos Investigators, the Brazilian Research in Intensive Care Network – BRICNET, the George Institute for Global Health and the ANZICS Clinical Trials Group.

Table 1S - Characteristics of fluid episodes in Brazil and in other countries

Variable	Brazil (n = 880)	Other countries (n = 1.836)	p value GEE
Crystalloid episode	843 (95.8)	1.365 (74.3)	< 0.0001
0.9% Sodium chloride	527 (62.5)	370 (27.1)	< 0.0001
Ringer's lactate/Ringer's acetate/Ringer's solution	270 (32.0)	537 (39.3)	0.064
Ringer's simple/Hartmann	29 (3.4)	148 (10.8)	< 0.0001
Plasmalyte	23 (2.7)	143 (10.5)	< 0.0001
Other balanced salt	0 (0.0)	140 (10.3)	< 0.0001
Other crystalloid	10 (1.2)	66 (4.8)	< 0.0001
Colloid episode	55 (6.2)	526 (28.6)	< 0.0001
Albumin	32 (58.2)	431 (81.9)	0.001
HES	18 (32.7)	33 (6.3)	< 0.0001
Gelatin	5 (9.1)	59 (11.2)	0.811
Dextran	0 (0.0)	4 (0.8)	< 0.0001

GEE - generalized estimating equation; HES - hydroxyethyl starch. Summary statistics of categorical variables are presented as percentages and p-values of the generalized estimating equation model (adjusted for patient-level clustering effect).

Table 2S - Fluid availability in intensive care units in Brazil (n = 217) and other countries (n = 209)

Fluid name	Brazil		Other countries	
	Yes	No	Yes	No
Crystalloid				
0.9% Saline	214 (98.6)	0 (0.0))	201 (96.2)	2 (1.0)
Hypertonic saline	139 (64.1)	72 (33.2)	132 (63.2)	70 (33.5)
Ringer's simple/Hartmann	146 (67.3)	67 (30.9)	50 (23.9)	152 (72.7)
Ringer's lactate	212 (97.7)	1 (0.5)	120 (57.4)	81 (38.8))
Plasmalyte A	15 (6.9)	190 (87.6)	18 (8.6)	183 (87.6)
Plasmalyte R	4 (1.8)	201 (92.6)	6 (2.9)	195 (93.3)
Plasmalyte 148 replacement	1 (0.5)	204 (94.0)	25 (12.0)	176 (84.2)
Ringer's acetate	2 (0.9)	203 (93.5)	58 (27.8)	143 (68.4)
Balanced glucose	3 (1.4)	202 (93.1)	40 (19.1)	161 (77.0)
Dextrose	211 (97.2)	2 (0.9)	162 (77.5)	40 (19.1)
Dextrose/saline	84 (38.7)	123 (56.7)	109 (52.2)	93 (44.5)
Hypertonic glucose	191 (88.0)	22 (10.1)	178 (85.2)	24 (11.5)
Colloid				
Albumin 4 - 5%	41 (18.9)	166 (76.5)	135 (64.6)	66 (31.6)
Albumin 20 - 25%	199 (91.7)	14 (6.5)	187 (89.5)	15 (7.2)
6% HES in saline	100 (46.1)	106 (48.8)	80 (38.3)	121 (57.9)
6% HES in balanced salt solution	27 (12.4)	178 (82.0)	34 (16.3)	166 (79.4)
Other starch	1 (0.5)	204 (94.0)	5 (2.4)	196 (93.8)
706 Plasma replacement	61 (28.1)	146 (67.3)	23 (11.0)	178 (85.2)
Gelatin	22 (10.1)	183 (84.3)	81 (38.8)	120 (57.4)
Haemaccel	44 (20.3)	161 (74.2)	14 (6.7)	187 (89.5)
Dextran 40 or 70	17 (7.8)	188 (86.6)	52 (24.9)	149 (71.3)

HES - hydroxyethyl starch. Results expressed as n (%).

Table 3S - Comparison of the characteristics of fluid use in 518 Brazilian patients in a total of 879 episodes by trauma status

Variable	Trauma		p value
	Yes<br>n = 50	No<br>n = 468	
Patients received crystalloid	50 (100.0)	456 (97.4)	0.252
Patients received colloid	2 (4.0)	36 (7.7)	0.341
Total volume of resuscitation fluid received on survey day (mL)	1000.0 (500.0 – 1.500.0)	1.000.0 (500.0 – 1.500.0)	0.217
Total volume of crystalloid received on survey day (mL)	1.000.0 (500.0 – 1.500.0)	1.000.0 (500.0 – 1.500.0)	0.298
Total volume of colloid received on survey day (mL)	275.0 (162.5 - 387.5)	275.0 (100.0 - 500.0)	0.505
Total volume of fluid input on the survey day (mL)	3.117.0 (2.347.5 – 4.490.8)	3.058.0 (2.000.0 – 4.164.0)	0.572
Total volume of fluid output on the survey day (mL)	1.400.0 (800.0 – 2.000.0)	1.380.0 (730.0 – 2.326.0)	0.952
Net fluid balance on the survey day (mL)	1.438.0 (772.0 – 2.549.0)	1.300.0 (500.0 – 2.515.0)	0.459
	**n = 90**	**n = 789**	**Valor de p**
Crystalloid episode	88 (97.8)	754 (95.6)	0.322
Colloid episode	2 (2.2)	53 (6.7)	0.095

Summary statistics of continuous variables are presented as the median (interquartile range), with p-values based on the nonparametric test (i.e., Wilcoxon rank-sum test). Summary statistics of categorical variables are presented as percentages, with p-values based on Pearson's Chi-squared test.

Table 4S - Comparison of characteristics of fluid use in 518 Brazilian patients in a total of 879 episodes by traumatic brain injury status

Variável	TBI		p value
	Yes<br>n = 14	No<br>n = 504	
Patients received crystalloid	14 (100.0)	492 (97.6)	0.559
Patients received colloid	0 (0.0)	38 (7.5)	0.286
Total volume of resuscitation fluid received on survey day (mL)	500.0 (500.0 – 1.500.0)	1.000.0 (500.0 – 1.500.0)	0.760
Total volume of crystalloid received on survey day (mL)	500.0 (500.0 – 1.500.0)	1.000.0 (500.0 – 1.500.0)	0.776
Total volume of colloid received on survey day (mL)	NA	275.0 (100.0 - 500.0)	NA
Total volume of fluid input on the survey day (mL)	3.663.3 (3.020.0 – 5.070.8)	3.054.0 (2.000.0 – 4.141.0)	0.064
Total volume of fluid output on the survey day (mL)	1.700.0 (925.0 – 2.450.0)	1.376.5 (745.0 – 2.306.2)	0.390
Net fluid balance on the survey day (mL)	1.478.0 (1.060.8 – 2.963.2)	1.312.0 (500.0 – 2.488.0)	0.237
	**n = 23**	**n = 856**	**Valor de p**
Crystalloid episode	23 (100.0)	819 (95.7)	0.308
Colloid episode	0 (0.0)	55 (6.4)	0.209

TBI - traumatic brain injury; NA – not applicable. Summary statistics of continuous variables are presented as the median (interquartile range), with p-values based on the nonparametric test (i.e., Wilcoxon rank-sum test). Summary statistics of categorical variables are presented as percentages, with p-values based on Pearson's Chi-squared test.

Table 5S - Comparison of characteristics of fluid use in 517 Brazilian patients in a total of 877 episodes by sepsis status

Variable	Sepsis		p value
	Yes<br>n = 205	No<br>n = 312	
Patients received crystalloid	201 (98.0)	304 (97.4)	0.651
Patients received colloid	16 (7.8)	22 (7.1)	0.748
Total volume of resuscitation fluid received on survey day (mL)	1.000.0 (500.0 – 1.500.0)	1.000.0 (500.0 – 1.500.0)	0.153
Total volume of crystalloid received on survey day (mL)	1.000.0 (500.0 – 1.500.0)	1.000.0 (500.0 – 1.500.0)	0.186
Total volume of colloid received on survey day (mL)	275.0 (100.0 - 623.5)	365.5 (100.0 - 500.0)	0.857
Total volume of fluid input on the survey day (mL)	3.311.0 (2.340.0 – 4.359.5)	2.903.0 (1.870.0 – 3.922.2)	0.004
Total volume of fluid output on the survey day (mL)	1.245.0 (565.0 – 2.062.5)	1.500.0 (820.0 – 2.425.0)	0.006
Net fluid balance on the survey day (mL)	1.705.5 (940.0 – 2.667.5)	1.187.0 (305.0 – 2.235.0)	<0.0001
	**n = 342**	**n = 535**	**Valor de p**
Crystalloid episode	328 (95.9)	512 (95.7)	0.883
Colloid episode	24 (7.0)	31 (5.8)	0.466

Summary statistics of continuous variables are presented as the median (IQR), with p-values based on the nonparametric test (i.e., Wilcoxon rank-sum test). Summary statistics of categorical variables are presented as percentages, with p-values based on Pearson's chi-squared test.

Table 6S - Comparison of characteristics of fluid use in 517 Brazilian patients in a total of 877 episodes by acute respiratory distress syndrome status

Variable	ARDS		p value
	Yes<br>n = 32	No<br>n = 485	
Patients received crystalloid	31 (96.9)	474 (97.7)	0.755
Patients received colloid	1 (3.1)	37 (7.6)	0.344
Total volume of resuscitation fluid received on survey day (mL)	1000.0 (500.0 - 1500.0)	1000.0 (500.0 - 1500.0)	0.261
Total volume of crystalloid received on survey day (mL)	1000.0 (500.0 - 1500.0)	1000.0 (500.0 - 1500.0)	0.129
Total volume of colloid received on survey day (mL)	150.0 (150.0 - 150.0)	300.0 (100.0 - 500.0)	NA
Total volume of fluid input on the survey day (mL)	3981.0 (2865.5 - 4988.5)	3016.0 (2000.0 - 4041.0)	0.003
Total volume of fluid output on the survey day (mL)	1000.0 (490.0 - 1925.0)	1400.0 (780.0 - 2350.0)	0.093
Net fluid balance on the survey day (mL)	2420.0 (1631.2 - 3758.6)	1285.0 (480.0 - 2464.0)	0.0001
	**n = 57**	**n = 820**	**Valor de p**
Crystalloid episode	54 (94.7)	786 (95.9)	0.685
Colloid episode	3 (5.3)	52 (6.3)	0.745

ARDS - acute respiratory distress syndrome; NA - not applicable. Summary statistics of continuous variables are presented as the median (interquartile range), with p-values based on the nonparametric test (i.e., Wilcoxon rank-sum test). Summary statistics of categorical variables are presented as percentages, with p-values based on Pearson's chi-squared test.

Table 7S - Univariate analysis of factors associated with the administration of crystalloids or colloids

Variable	OR (95%CI) for receiving crystalloid	p value	OR (95%CI) for receiving colloid	p value
Age (per 1-year increase)	1.0 (1.0 - 1.0)	0.709	1.0 (1.0 - 1.0)	0.817
Sex, male	0.9 (0.4 – 2.1)	0.813	0.8 (0.4 – 1.6)	0.541
Number of days in the ICU at survey date	1.0 (1.0 - 1.0)	0.204	1.0 (1.0 - 1.0)	0.740
Number of days in the ICU at survey date > 0	0.4 (0.1 - 1.3)	0.120	1.4 (0.6 - 3.3)	0.486
Admission characteristics*				
Operating room after elective surgery	1.0	0.268	1.0	0.085
Emergency room	1.1 (0.3 - 3.4)		0.5 (0.2 - 1.5)	
Hospital floor	3.9 (0.5 - 31.9)		0.2 (0.04 - 0.8)	
Transferred from other ICU or hospital	3.2 (0.4 - 26.7)		0.6 (0.1 - 2.8)	
Operating room after emergency surgery	0.7 (0.2 - 2.4)		0.9 (0.3 - 2.5)	
Hospital floor after previous ICU stay	0.4 (0.1 - 1.5)		1.8 (0.7 - 4.8)	
Surgical admission diagnosis	0.7 (0.3 - 1.7)	0.423	1.6 (0.8 - 3.2)	0.194
Trauma at hospital admission	1.7 (0.4 - 7.4)	0.455	0.3 (0.1 - 1.4)	0.133
Sepsis in 24 hours prior to survey date	1.2 (0.5 - 2.8)	0.745	1.2 (0.6 - 2.4)	0.612
ARDS in 24 hours prior to survey date	1.0 (0.1 - 7.8)	0.987	0.6 (0.1 - 4.3)	0.585
APACHE II chronic health points criteria (any)	1.0 (0.4 - 2.4)	0.924	1.2 (0.6 - 2.6)	0.631
Indication for fluid in each fluid resuscitation				
Impaired perfusion/low cardiac output	1.0	0.479	1.0	0.714
Abnormal vital signs	1.3 (0.5 - 3.7)		0.6 (0.2 - 1.5)	
Ongoing bleeding/other fluid losses	0.5 (0.2 - 1.8)		1.1 (0.4 - 3.2)	
Unit protocol/other	0.4 (0.1 - 2.6)		1.3 (0.2 - 7.5)	
Fluid prescriber*				
Specialist	1.0	0.026	1.0	0.140
Senior resident/fellow	3.8 (0.7 - 19.5)		0.4 (0.1 - 1.4)	
Resident	0.2 (0.1 - 0.9)		2.4 (0.7 - 8.6)	
Cardiac failure (cardiac SOFA ≥ 3)	0.8 (0.4 - 1.3)	0.336	0.9 (0.5 - 1.8)	0.829
Respiratory failure (respiratory SOFA ≥ 3)	0.8 (0.3 - 2.1)	0.711	1.6 (0.8 - 3.5)	0.200
Renal replacement therapy	1.4 (0.3 - 6.5)	0.659	1.6 (0.6 - 4.3)	0.300
Mechanical ventilation	1.0 (0.4 - 2.9)	0.946	1.2 (0.7 - 2.2)	0.563
Metabolic acidosis*	0.4 (0.1 - 1.6)	0.007	1.5 (0.6 - 3.4)	0.031
Metabolic acidosis. Missing	0.2 (0.0 - 0.5)		2.4 (1.2 - 4.8)	
Lactate ≥ 2mmol/L*	1.7 (0.2 - 11.9)	0.058	0.7 (0.3 - 2.1)	0.240
Lactate (mmol/L). Missing	0.4 (0.1 - 1.5)		1.4 (0.6 - 3.1)	
Heart rate, b/minute	1.0 (1.0 - 1.0)	0.679	1.0 (1.0 - 1.0)	0.322
Heart rate (per 10 b/minute increase)	1.0 (0.9 - 1.2)	0.842	1.1 (1.0 - 1.2)	0.168
Mean arterial pressure*	1.0 (1.0 - 1.0)	0.060	1.0 (1.0 - 1.0)	0.083
Mean arterial pressure (per 10mmHg decrease)*	1.2 (1.0 - 1.4)	0.059	0.9 (0.7 - 1.0)	0.041
Creatinine ≥ 170 (μmol/L)	0.8 (0.2 - 2.5)	0.672	1.2 (0.5 - 3.0)	0.747
Bilirubin ≥ 2.0 (μmol/L) categories†	0.0 (0.0 - Inf)	< 0.0001	0.0 (0.0 - 0.0)	< 0.0001
Albumin ≥ 27g/L*	4.8 (0.1 - 401.0)	0.427	0.2 (0.02 - 1.7)	0.006
Albumin, missing	2.0 (0.6 - 6.6)		0.3 (0.1 - 0.6)	
Urine output ≥ 0.5mL/kg/hour	0.4 (0.1 - 1.2)	0.261	1.0 (0.5 - 2.1)	0.859
Urine output, missing	0.5 (0.1 - 2.3)		1.2 (0.5 - 3.0)	
Fluid output ≥ 1mL/kg/hour	1.5 (0.7 - 2.9)	0.323	0.9 (0.4 - 1.9)	0.829
Fluid output missing	1.7 (0.6 - 4.6)		0.8 (0.4 - 1.7)	

RC - razão de chance; IC95% - intervalo de confiança de 95%; UTI - unidade de terapia intensiva; SDRA – síndrome do desconforto respiratório agudo; APACHE - *Acute Physiology and Chronic Health Evaluation*; SOFA - *Sequential Organ Failure Assessment*. Variáveis categóricas com 20% de valores faltantes foram analisadas como dados faltantes apenas como grupos separados. Os resultados foram gerados de modelos de equações de estimativas generalizadas com as identidades dos pacientes atribuídas como agrupamentos. As quantidades de dados disponíveis para "traumatismo cranienceférico", "categoria de trauma na admissão ao hospital", "baixa pressão de enchimento" e "bilirrubina" foram muito pequenas. Assim, essas variáveis foram excluídas da análise multivariada independentemente dos baixos valores de p nos modelos de equações de estimativas generalizadas. "APACHE II pontos", "indicação para fluidos em cada episódio", e "prescritor dos fluidos" precisaram ser reagrupados na análise univariada em razão do pequeno tamanho da amostra em algumas subcategorias. *Variáveis com valores de p nas equações de estimativas generalizadas < 0,1 foram salientadas e elegíveis para inclusão na análise multivariada, † embora com p < 0,1, em razão da distribuição extremamente desigual das categorias de bilirrubina entre os grupos de episódios com cristaloides e coloides Sim/Não, os resultados (razão de chance e intervalo de confiança de 95%) são altamente não confiáveis. Assim, a variável de categoria de bilirrubina NÃO foi incluída na análise multivariada.
